# Association between daptomycin susceptibility and teicoplanin resistance in *Staphylococcus epidermidis*

**DOI:** 10.1038/s41598-019-55149-z

**Published:** 2019-12-06

**Authors:** Shinichi Watanabe, Yukinobu Kawakami, Hiroshi Kimura, Shinobu Murakami, Hitoshi Miyamoto, Shingo Takatori, Koichiro Suemori, Mamoru Tanaka, Akihiro Tanaka, Keiko Tanaka, Hisamichi Tauchi, Jun Maki, Hiroaki Araki, Takumi Yamaguchi

**Affiliations:** 1 Department of Clinical Pharmacy, College of Pharmaceutical Sciences, Matsuyama University, 4-2 Bunkyo-cho, Matsuyama, Ehime 790-8578 Japan; 2 Division of Pharmacy, Ehime University Hospital, 454 Shitsukawa, Toon, Ehime 791-0295 Japan; 3 Department of Infection Control, Ehime University Hospital, 454 Shitsukawa, Toon, Ehime 791-0295 Japan; 4 Department of Epidemiology and Preventive Medicine, Ehime University Graduate School of Medicine, 454 Shitsukawa, Toon, Ehime 791-0295 Japan; 5 Research Promotion Unit, Translation Research Center, Ehime University Hospital, 454 Shitsukawa, Toon, Ehime 791-0295 Japan; 6 Department of Infectious Disease, College of Pharmaceutical Sciences, Matsuyama University, 4-2 Bunkyo-cho, Matsuyama, Ehime 790-8578 Japan; 7 School of Pharmacy, Shujitsu University, 1-6-1 Nishigawara, Naka-ku, Okayama, Okayama, 703-8516 Japan

**Keywords:** Bacterial infection, Risk factors

## Abstract

*Staphylococcus epidermidis* infections are a common occurrence in hospitals, particularly in catheter-related bloodstream and surgical site infections and infective endocarditis. Higher daptomycin minimum inhibitory concentration (MIC) values may be associated with daptomycin treatment failure among patients with *S. epidermidis* infections. We therefore conducted a retrospective cohort study to determine the predictive value of daptomycin susceptibility. A retrospective study was undertaken in 1,337 patients with *S. epidermidis* infections. Data were collected from 1 January 2013 to 31 December 2016 at Ehime University Hospital, and included the following clinicopathological factors for evaluation: age, sex, resistance to vancomycin or teicoplanin, and history of antimicrobial therapy. Multiple analysis was performed using logistic regression to identify factors that independently and significantly affected the daptomycin resistance. Daptomycin-resistant *S. epidermidis* was identified in 38 (2.8%) patients. According to the multiple analysis, only higher MIC values (≥16 mg/L) for teicoplanin (*P* < 0.0001) were independently associated with an increased risk of developing daptomycin resistance. In conclusion, higher teicoplanin MIC values may predict resistance to daptomycin treatment in *S. epidermidis* infections.

## Introduction

Daptomycin, a cyclic polypeptide and a semisynthetic lipopeptide antibiotic, is derived from *Streptomyces roseosporus*^1^. The native molecule is anionic, although binding to the bacterial membrane and exploitation of the bactericidal activity that is typical of daptomycin require calcium. Daptomycin exhibits bactericidal activity by penetrating the bacterial cell wall and binding to the cytoplasmic membrane, thereby causing rapid depolarization of the membrane. This results in the loss of membrane potential and bacterial cell death^2^. Because of the unique mechanism of action, daptomycin-resistance has rarely been documented. However, treatment failure due to daptomycin resistance was observed in a patient with high-grade methicillin-sensitive *Staphylococcus aureus* (MRSA) bacteraemia^3,4^.

*Staphylococcus epidermidis* infections, particularly catheter-related bloodstream and surgical site infections and infective endocarditis, are common in hospitals^5,6^. *S. epidermidis* mostly engages beneficially in the host defence and immune maturation system but may show infective behaviour under certain circumstances^7^. Although a reduction in the susceptibility of *S. aureus* to daptomycin is well established, that of *S. epidermidis* is unknown. A high minimum inhibitory concentration (MIC) of daptomycin may contribute to treatment failure in patients with *S. epidermidis*, especially methicillin-resistant *S. epidermidis* (MRSE), infections. We, therefore, conducted a retrospective cohort study to determine the predictive value of daptomycin susceptibility.

## Results

The data from 1,337 patients were included in this study, of whom, 1,299 (97.2%) had a lower daptomycin MIC (≤1 mg/L) for *S. epidermidis* and 38 (2.8%) had a higher daptomycin MIC (MIC >1 mg/L) for *S. epidermidis*. These patients were categorized into low (≤1 mg/L; sensitive) and high (>1 mg/L; resistant) MIC groups. A comparison of the demographic characteristics between the two groups is shown in Table 1. The groups were equivalent with respect to age, sex, implantation, vancomycin susceptibility, and prior use of linezolid, cephem, aminoglycoside, fluoroquinolone, and sulfamethoxazole/trimethoprim. However, there were significant differences in terms of prior use of glycopeptide (*P* = 0.003), daptomycin (*P* = 0.018), penicillin (*P* = 0.016), carbapenem (*P* = 0.014), and tetracycline (*P* = 0.016), as well as teicoplanin susceptibility (*P* < 0.0001).

**Table 1:** Patient demographics.

Characteristics	Daptomycin susceptibility		*P*-value
	Sensitive (%)	Resistant (%)	
Total	1299 (97.2)	38 (2.8)	
**Age**			
<65	632 (48.7)	18 (47.4)	0.876
≥65	667 (51.4)	20 (52.6)	
**Sex (male/female)**			
Male	772 (59.4)	24 (63.2)	0.645
Female	527 (40.6)	14 (36.8)	
**Implantation, catheter**			
With	223 (17.2)	11 (28.9)	0.060
Without	1076 (82.8)	27 (71.1)	
**Previous glycopeptide use**			
Yes	305 (23.5)	17 (44.7)	0.003
No	994 (76.5)	21 (55.3)	
**Vancomycin susceptibility**			
MIC < 2 mg/L	1016 (78.2)	27 (71.1)	0.293
MIC ≥ 2 mg/L	283 (21.8)	11 (28.9)	
**Teicoplanin susceptibility**			
MIC < 16 mg/L	1212 (93.3)	23 (60.5)	<0.0001*
MIC ≥ 16 mg/L	87 (6.7)	15 (39.5)	
**Previous linezolid use**			
Yes	29 (2.2)	2 (5.3)	0.219
No	1270 (97.8)	36 (94.7)	
**Previous daptomycin use**			
Yes	91 (7.0)	7 (18.4)	0.018*
No	1208 (93.0)	31 (81.6)	
**Previous penicillin use**			
Yes	469 (36.1)	21 (55.3)	0.016
No	830 (63.9)	17 (44.7)	
**Previous cephem use**			
Yes	863 (66.4)	28 (73.7)	0.350
No	436 (33.6)	10 (26.3)	
Previous carbapenem use			
Yes	497 (38.3)	22 (57.9)	0.014
No	802 (61.7)	16 (42.1)	
**Previous aminoglycoside use**			
Yes	97 (7.5)	3 (7.9)	0.921
No	1202 (92.5)	35 (92.1)	
**Previous tetracycline use**			
Yes	68 (5.2)	6 (15.8)	0.016*
No	1231 (94.8)	32 (84.2)	
**Previous fluoroquinolone use**			
Yes	659 (50.7)	24 (36.8)	0.131
No	640 (49.3)	14 (63.2)	
**Previous sulfamethoxazole/trimethoprim use**			
Yes	237 (18.2)	7 (18.4)	0.978
No	1062 (81.8)	31 (81.6)	

*The Fisher’s exact test.

The relationships between the study variables and daptomycin resistance are shown in Table 2. In the crude logistic regression analysis, a higher teicoplanin MIC value (*P* < 0.0001) and prior use of glycopeptide (*P* = 0.005), daptomycin (*P* = 0.023), penicillin (*P* = 0.018), carbapenem (*P* = 0.016), and tetracycline (*P* = 0.020) were associated with increased risk of developing daptomycin resistance. In the multiple analysis, only higher teicoplanin MIC value was independently associated with an increased risk of daptomycin resistance (adjusted OR = 8.01, 95% CI = 3.86–16.18, *P* < 0.0001). Additionally, the MIC of daptomycin was significantly positively correlated with that of teicoplanin (Pearson correlation coefficient (r) = 0.55, *P* < 0.0001).

**Table 2:** Relationships between the studied variables and daptomycin resistance.

Variables	Crude OR (95% CI)	*P*-value	Adjusted OR (95% CI)*	*P*-value
Age (≥65)	1.05 (0.55–2.03)	0.876	1.25 (0.67–2.46)	0.518
Sex (male/female)	1.17 (0.61–2.34)	0.643	1.07 (0.55–2.18)	0.844
Implantation, catheter	1.97 (0.92–3.92)	0.078		
Previous glycopeptide use	2.64 (1.36–5.06)	0.005	1.91 (0.95–3.77)	0.070
Higher vancomycin MIC value (MIC ≥2 mg/dL)	1.46 (0.69–2.91)	0.309		
Higher teicoplanin MIC value (MIC ≥16 mg/dL)	9.09 (4.49–17.90)	<0.0001	8.01 (3.86–16.18)	<0.0001
Previous linezolid use	2.43 (0.38–8.52)	0.290		
Previous daptomycin use	3.00 (1.19–6.62)	0.023		
Previous penicillin use	2.19 (1.14–4.24)	0.018		
Previous cephem use	1.41 (0.70–3.09)	0.341		
Previous carbapenem use	2.22 (1.16–4.34)	0.016		
Previous aminoglycoside use	1.06 (0.25–3.02)	0.922		
Previous tetracycline use	3.40 (1.25–7.86)	0.020		
Previous fluoroquinolone use	1.67 (0.87–3.33)	0.128		
Previous sulfamethoxazole/trimethoprim use	1.01 (0.41–2.20)	0.978		

*Simultaneous adjustment of each variable that was significant (*P* < 0.01) in the crude model.

## Discussion

Our findings demonstrate that a lower susceptibility to teicoplanin was independently associated with an increased risk of lower susceptibility to daptomycin in patients with *S. epidermidis* infection. Teicoplanin, a glycopeptide, currently used for the treatment of potentially serious infections caused by β-lactam-resistant gram-positive pathogens, is not approved in the United States. However, in Europe, Japan, and Taiwan, it is commonly prescribed as vancomycin. Teicoplanin is considered as effective as vancomycin for the treatment of MRSA bacteraemia^8^. In cases of MRSE infection, glycopeptides or daptomycin is used for the treatment of MRSA.

Daptomycin resistance has been reported in cases of bacteraemia, endocarditis, osteomyelitis, skin and soft-tissue infections, and prosthetic joint infection. Where daptomycin non-susceptibility developed, other antibiotics were used previously (mainly vancomycin or teicoplanin)^4^. Thus, a vast majority of cases of daptomycin resistance were reported in patients previously treated with a glycopeptide antibiotic. The heterogeneous vancomycin-intermediate *S. aureus* (hVISA) with a vancomycin MIC of 1–2 mg/L were resistant to daptomycin. However, a higher MIC (≥2 mg/L) of vancomycin or other antimicrobials did not affect daptomycin resistance in this study. The majority of previous reports did not describe the mechanism of daptomycin resistance. A previous study on vancomycin resistance in VISA strains, however, indicated that a thickened cell wall was a common characteristic among the VISA strains, serving as a physical barrier against the penetration of vancomycin molecules and resulting in vancomycin resistance^9,10^. Furthermore, Garcia *et al*. reported that MRSE was the leading cause of coagulase-negative staphylococcal infective endocarditis, and patients receiving vancomycin had higher mortality rates than those receiving cloxacillin. The mortality was higher among patients having isolates with a vancomycin MIC of ≥2 mg/L^11^. Several mechanisms contributing to the reduced susceptibility of Staphylococci to daptomycin have been reported, many of which describe a common resistance mechanism of *S. aureus* and *S. epidermidis* to antibiotics^4,12^. The mechanism of *S. epidermidis* resistance is similar to that of VISA and hVISA strains^13,14^. A possible explanation of this correlation is that the thickened cell wall acts as a common barrier to daptomycin and teicoplanin penetration, although daptomycin does not bind to peptidoglycan to form a subsequent physical barrier within the cell wall^15,16^. As daptomycin (molecular weight 1620.67 Da) and teicoplanin (molecular weight 1564.25–1893.68 Da) are relatively larger in molecular size as compared to vancomycin (molecular weight 1485.7 Da), we suggest that daptomycin might not be able to efficiently penetrate the cell wall if it is as thick as that of *S. epidermidis*. In this case, daptomycin might be impeded by the thickened cell wall before reaching the cytoplasmic membrane resulting in ineffective bactericidal function in the target cells.

Furthermore, the ability of *S. epidermidis* to produce biofilms ensures significant resistance to antibiotics and impairs the host innate immune response^6^. In these situations, the use of an antimicrobial agent capable of crossing the biofilm enhances the treatment efficacy. In this context, the ability of daptomycin to penetrate rapidly into the biofilm produced by *S. epidermidis* is well known^17^. However, daptomycin treatment is not suitable for daptomycin-resistant *S. epidermidis* infection. In this study, patients with bloodstream infections caused by daptomycin resistant *S. epidermidis* were mainly treated with vancomycin. Hence, there was no difference in the treatment duration between the sensitive (12.2 days) and resistant (13.0 days) groups. There are some limitations to our study, including the retrospective design. Furthermore, genetic analysis of *S. epidermidis* was not performed. Due to the very small number of patients in the resistance group (n = 38), our confidence interval was wide which provides a lesser statistical certainty, and hence, the positive significant results might have happened by chance.

In conclusion, a higher teicoplanin MIC may predict the resistance of *S. epidermidis* to daptomycin. A more effective therapeutic strategy against daptomycin-resistant *S. epidermidis* bacteraemia, such as initial regimens that combine different classes of antimicrobial agents or early transition to an alternative treatment, is required. The true significance of the clinical activity of daptomycin against teicoplanin-resistant *S. epidermidis* warrants further explanation and investigation in future clinical studies.

## Patients and Methods

### Ethical approval of the study protocol

The study was carried out in accordance with the guidelines on human studies adopted by the ethics committee of Ehime University Hospital (Ehime, Japan; review board approval no. 1602008), the Declaration of Helsinki, and the Ethical Guidelines on Medical and Health Research Involving Human Subjects by the Ministry of Education, Culture, Sports, Science and Technology, and the Ministry of Health, Labour and Welfare of Japan. The Japanese law does not mandate obtaining individual informed consent from participants in non-invasive observational trials, such as the present study. Therefore, we used our official pharmacy website as an opt-out method rather than obtaining written or verbal informed consent from each patient.

### Patients and study design

The data were collected from 1 January 2013 to 31 December 2016 at Ehime University Hospital. In this study, a total of 1699 patients with *S. epidermidis* infection were included and 362 patients were excluded due to missing data on daptomycin MIC values. The data of 1337 patients with *S. epidermidis* infection available for analysis in a retrospective study (participation rate, 78.7%). As compared to the excluded patients, the study patients were more likely to use glycopeptide, daptomycin, and cephem, and less likely to show a higher teicoplanin susceptibility (MIC ≥16 mg/L) or use fluoroquinolone and sulfamethoxazole/trimethoprim. The following clinicopathological factors were obtained from the medical charts: age, sex, implantation, resistance to vancomycin or teicoplanin, and history of antimicrobial therapy. Where one or more samples were determined as resistant, the patients were included in the resistant group. The criteria for MIC value were according to the CLSI M100 S28 guidelines. According to the CLSI guidelines, an MIC of ≤4 mg/L signifies sensitivity to vancomycin. However, a low success rate of vancomycin therapy with a vancomycin MIC of ≥2 mg/L has previously been reported^18,19^. Therefore, patients with a vancomycin MIC of ≥2 mg/L were included in the lower susceptibility group. The determination of MIC was carried out using the standard broth microdilution method.

### Statistical analysis

 χ^2^-test and Fisher’s exact test were used to compare the categorical variables. Using logistic regression analysis, we calculated the crude and adjusted odds ratios (ORs) with their 95% confidence intervals (CIs). Parameters with a *P*-value of < 0.01 in the crude analyses were considered for inclusion in the multivariate model. All statistical analyses were performed using the SAS software package version 9.4 (SAS Institute, Inc., NC, USA).

## Supplementary information

Supplementary material
