# Supplementary material for: Association between daptomycin susceptibility and teicoplanin resistance in Staphylococcus epidermidis
Source: Sci Rep. 2019 Dec 6;9:18533. doi: 10.1038/s41598-019-55149-z (PMC6898446; doi:10.1038/s41598-019-55149-z)
Supplement: Supplementary file 1 — Supplementary material [file 41598_2019_55149_MOESM1_ESM.docx]

**Association between daptomycin susceptibility and teicoplanin resistance in *Staphylococcus epidermidis***

Shinichi Watanabe^1#^, Yukinobu Kawakami^2^, Hiroshi Kimura^2,3^, Shinobu Murakami^3^, Hitoshi Miyamoto^3^, Shingo Takatori^1^, Koichiro Suemori^3^, Mamoru Tanaka^2^, Akihiro Tanaka^2^, Keiko Tanaka^4,5^, Hisamichi Tauchi^3^, Jun Maki^6^ , Hiroaki Araki^7^ and Takumi Yamaguchi^1^

***^1^Department of Clinical Pharmacy, College of Pharmaceutical Sciences, Matsuyama University, 4-2 Bunkyo-cho, Matsuyama, Ehime 790-8578, Japan***

***^2^Division of Pharmacy, Ehime University Hospital,* *454 Shitsukawa, Toon, Ehime 791-0295, Japan***

*^3^****Department of Infection Control, Ehime University Hospital, 454 Shitsukawa, Toon, Ehime 791-0295, Japan***

***^4^Department of Epidemiology and Preventive Medicine, Ehime University Graduate School of Medicine, 454 Shitsukawa, Toon, Ehime 791-0295, Japan***

***^5^Research Promotion Unit, Translation Research Center, Ehime University Hospital, 454 Shitsukawa, Toon, Ehime 791-0295, Japan***

***^6^Department of Infectious Disease, College of Pharmaceutical Sciences, Matsuyama University, 4-2 Bunkyo-cho, Matsuyama, Ehime 790-8578, Japan***

***^7^School of Pharmacy, Shujitsu University, 1-6-1 Nishigawara, Naka-ku, Okayama, Okayama 703-8516, Japan***

Running Head: Daptomycin resistance in *Staphylococcus epidermidis*

#Address correspondence to Shinichi Watanabe

Department of Clinical Pharmacy, College of Pharmaceutical Sciences, Matsuyama University, 4-2 Bunkyo-cho, Matsuyama, Ehime 791-0295, Japan

E-mail: swatanab@g.matsuyama-u.ac.jp

Tel.: +81-89-926-7113

Fax: +81-89-922-7162

**Supplementary material**

**C**orrelation between the MIC of daptomycin and that of teicoplanin.
